# Supplementary material for: Research on improved gangue target detection algorithm based on Yolov8s
Source: PLoS One. 2024 Jul 9;19(7):e0293777. doi: 10.1371/journal.pone.0293777 (PMC11232985; doi:10.1371/journal.pone.0293777)
Supplement: Attachment — Submitted filename: new Response to Reviewers.docx [file pone.0293777.s002.docx]

Response to Reviewer 1

Thank you for your review of our paper.

Response to Reviewer 2

Thank you for your review of our paper. We have answered each of your points below.

1. [I wonder why the new version has much more line space than last version. Please ensure you are using correct template.]

Response: I found in the submission instructions "The manuscript text should be double-spaced", so the correct template is being used. The manuscript text is double-spaced.

Response to Reviewer 2

Thank you for your review of our paper. We have answered each of your points below.

1. [You should clarify who are the annotators, do you have annotation agreement between annotators?]

Response: I'm sorry maybe I didn't think through your question, the dataset question was done by me at the time, there were no other annotators and no other disagreements between authors.
